# Defining the minimally clinically important difference of the SF-36 physical function subscale for paediatric CFS/ME: triangulation using three different methods

**DOI:** 10.1186/s12955-018-1028-2

**Published:** 2018-10-19

**Authors:** Amberly Brigden, Roxanne M Parslow, Daisy Gaunt, Simon M Collin, Andy Jones, Esther Crawley

**Affiliations:** 1 Population Health Sciences, Centre for Academic Child Health, Bristol Medical School, University of Bristol, 1-5 Whiteladies Road, Bristol, BS8 1NU UK; 2 Bristol Randomised Trials Collaboration, Population Health Sciences, Bristol Medical School, University of Bristol, Bristol, BS8 2PS UK

**Keywords:** Minimal clinically important difference (MCID), Paediatric, Chronic fatigue syndrome, Myalgic encephalomyelitis, CFS/ME

## Abstract

**Background:**

Defining the minimally clinically important difference (MCID) is important for the design and analysis of clinical trials and ensures that findings are clinically meaningful. Studies in adult populations have investigated the MCID of The Short Form 36 physical function sub-scale (SF-36-PFS). However, to our knowledge no studies have defined the MCID of the SF-36-PFS in a paediatric population. We aimed to triangulate findings from distribution, anchor and qualitative methods to identify the MCID of the SF-36-PFS for children and adolescents with CFS/ME.

**Methods:**

Quantitative methods: We analysed routinely-collected data from a specialist paediatric CFS/ME service in South-West England using: 1) the anchor method, based on Clinical Global Impression (CGI) outcomes at 6 months’ follow-up; 2) the distribution method, based on the standard deviation of baseline SF-36-PFS scores.

Qualitative methods: Young people (aged 12–17 years) and parents were asked to complete the SF-36-PFS, marking each question twice: once for where they would currently rate themselves/their child and a second time to show what they felt would be the smallest amount of change for them/their child to feel treatment had made a difference. Semi-structured interviews were designed to explore what factors were deemed important to patients and to what extent an improvement was considered satisfactory. We thematically analysed qualitative interviews from 21 children and their parents.

**Results:**

Quantitative results: Six-month follow-up data were available for 198 children with a mean age of 14 years. Most were female (74%, 146/198) and 95% gave their ethnicity as “White British”.

Half the standard deviation of the baseline SF-36-PFS scores was 11.0. “A little better” on the CGI equated to a mean difference on the SF-36-PFS from baseline to 6-month follow-up of 9.0.

Qualitative results: Twenty-one children with CFS/ME participated: 16 females (76.2%) with a mean age of 14.4 years. Twenty mothers and two fathers were also interviewed.

The median minimal improvement in the SF-36-PFS was 10. Participants indicated that small changes in physical function can lead to important improvements in valued social and family function. Patients and parents were positive about improvement even in the presence of persisting symptoms.

Triangulation: The MCID based on the mean score from the three methods was 10.

**Conclusions:**

Converging evidence indicates future studies in paediatric CFS/ME should use an MCID of 10 on the SF-36-PFS.

**Electronic supplementary material:**

The online version of this article (10.1186/s12955-018-1028-2) contains supplementary material, which is available to authorized users.

## Background

The Minimal Clinically Important Difference (MCID) is defined as “the smallest difference in score in the domain of interest which patients perceive as beneficial and which would mandate, in the absence of troublesome side effects and excessive cost, a change in the patient’s management” [1]. The MCID is used in trials to calculate sample size and interpret results [2]. Distribution methods compare the change in scores on the outcomes measure to a measure of variability, including the standard error of measurement (SEM), the standard deviation (SD), the effect size, or the minimum detectable change (MDC) [3]. Whilst a variety of effect sizes and SEMs can be used, calculating the MCID using 0.5 SD is popular and it has been show to corresponds to the MCID across a variety of studies [4]. However, distribution methods may not be clinically relevant to patients [5] and will vary on the sample size that the SD is based on. Alternative methods to determining a more clinically relevant MCID are the anchor method and using qualitative methodology. The anchor method correlates the change on an outcome measure with prospectively collected change data on a global assessment scale [6] – i.e. those who defined the difference as “better” are compared against those who stated they were “unchanged”. However, there are limitations of the anchor method. The decision about where the cut-off should be on the anchor scale is often arbitrary [7] and arguably, a global assessment may not always be valid [8]. Qualitative methods enable richer exploration of the patient’s perspective of minimal level of change, but can lack the precision needed to determine a numerical marker of MCID [8]. Table 1 summarise the advantages and disadvantages of these methods. Because of the strengths and limitations of each method, triangulating multiple methods provides a solution to defining a precise and clinically meaningful MCID [8].

**Table 1:** A summary of the advantages and disadvantage of distribution, anchor and qualitative methods

	Advantages	Disadvantages
Distribution method	• Distribution methods are based on statistical models [3]. • The value of 0.5 SD corresponds to the MCID across a variety of studies [4].	• Guidelines for the interpretation of effect size are somewhat arbitrary. • This statistical approach does not consider the core concept of the MCID; the clinical importance [8]. • These methods are sample-specific; findings will vary on the sample size and distribution that the SD is based on [38].
Anchor method	• Anchor methods have the advantage of being more clearly understood because change scores are related to a clearly understood clinical observation [39]. • Global assessment scales are sensitive to change [40].	• Determining the cut-off on the anchor scale is often an arbitrary decision [7]. • Global assessment scales may not always be valid. For example, they can be susceptible to recall bias [41].
Qualitative methods	• Gathering the views and experiences of patients provides clinical relevance to the MCID. • Qualitative data provides richer information from the participants perspective which cannot be elicited through standardized measures [8].	• Can lack the precision needed to determine a numerical marker of MCID [8]. • Often includes smaller sample sizes, which can introduce issues with generalisability [42].

The Short Form 36 (SF-36) [9] is a widely used general health-status measure. The SF-36 physical function sub-scale (SF-36-PFS) consists of ten items and is used to measure change in disability in many chronic illnesses including Chronic Fatigue Syndrome/Myalgic Encephalomyelitis (CFS/ME) [10–13]. An MCID defined as 0.3/0.5 SD of the SF-36-PFS, typically equating to 8 to 10 points has been used in adult and paediatric CFS/ME [14–17], but it is unclear if this is appropriate and clinically relevant for patients. In other conditions, the Delphi method has suggested an MCID of 10 for asthma and heart disease [18], and 5 for Chronic Obstructive Pulmonary Disease in adults [18]. Anchor and distribution methods have identified the MCID as ranging from 3.25–20.40 for hip and knee replacement [19] and 7.1 for rheumatoid arthritis [20]. However, extrapolating findings from other clinical populations is inappropriate [18, 19]. We are not aware of any studies that have attempted to identify the clinically relevant MCID for children.

In this study, we used three methods to define the MCID of the SF-36-PFS in paediatric CFS/ME, a relatively common (prevalence 0.4–2.4% [21–24]) and disabling condition [25, 26]. We used the distribution method, the anchor method, and a qualitative method.

## Methods

### The SF-36-PFS

The SF-36 is the most widely used general health-status measure [27]. It includes eight scales, including the physical subscale which captures functional impairment. The SF-36-PFS has 10 items and scoring ranges from 0 to 100, with higher scores indicating better physical function. This subscales asks respondents how limited they are (“a lot”, “a little”, “not at all”) on everyday activities (e.g. “Bathing or dressing yourself”). The SF-36 has been shown to be reliable and valid with acceptable internal consistency coefficients and differentiate psychiatric patients, patients with minor conditions and chronic diseases [28].

### Anchor method and distribution methods

#### Participants

We analysed routinely-collected data from a specialist paediatric CFS/ME service in South-West England. Participants completed the measures on paper forms which were returned to the clinical team by hand at assessment and by post at follow- up appointments. These included the SF-36-PFS [9] collected at baseline and 6-month follow-up and the Clinical Global Impression (CGI) scale collected at 6 month follow-up. The CGI consists of one item: “Overall, how much have you changed since you first came to the service?”, and is scored from 1 to 7, with 1 indicating “Very much better”, 2 “Much better”, 3 “A little better”, 4 “No change”, 5 “A little worse”, 6 “Much worse”, 7 “Very much worse”.

#### Analyses

##### Distribution method

Half of the standard deviation of the baseline SF-36-PFS score at assessment was used to calculate the MCID [4].

##### Anchor method

We calculated the mean difference (and 95% CI) between baseline and 6-month follow-up scores for each level of response on the CGI.

### Qualitative methods

Data were used from a larger qualitative study exploring how “recovery” should be measured in paediatric CFS/ME, and what improvement in fatigue and disability are important to young people and their parents. The participants from this study were young people with CFS/ME and their families, recruited from the Royal United Hospitals, Bath Specialist Paediatric CFS/ME service. This service provides assessment and treatment to over 400 children/adolescents with CFS/ME annually. Young people were eligible if they were: diagnosed with CFS/ME using NICE guidelines [29], mild to moderately affected (not housebound) and aged between 12 and 17 years.

#### Qualitative interviews procedure

A section of the semi-structured topic guide (lasting 30–45 min) included questions on the MCID; young people and their parent/carers were asked open-ended questions designed to explore what they considered the smallest change on each item of the SF-36-PFS to be subjectively meaningful. During the semi-structured interview, children were asked to complete the SF-36-PFS. Participants were asked to mark each question twice: once for where they would currently rate themselves and a second time to show what they felt would be the smallest amount of change for them to feel treatment had made a difference which was worth having treatment for. Parents/carers were also asked to complete this process; to mark their child’s current health status and then provide a second mark to indicate the smallest amount of change to feel like treatment has made a difference for their child. See Additional file 1 for the topic guide.

Most interviews were undertaken in the participant’s own home, one interview was undertaken on hospital premises to coincide with an appointment. Participants included those recently assessed in clinic and those from a year ago to understand how the impact changes with illness duration. Children and their parent(s) were mostly interviewed separately except in 4 out of 21 interviews a parent was present in the room at the time of the child’s interview. The transcript section regarding MCID was not available for 1 child and 3 parents.

Each interview was audio recorded, transcribed, anonymised and imported into NVivo. Two researchers analysed the transcripts using thematic analysis [30].

## Results

### Quantitative results

Six-month follow-up data were available for 198 children, representing 26.9% (198/737) of children for whom baseline data were available. Participants were aged between six and 17 years, with a mean age of 14 (SD 2.3). Most were female (74%, 146/198) and 95% (185/195) gave their ethnicity as “White British”. Mean (SD) baseline and 6-month follow-up SF-36-PFS scores were 51.5 (21.4) and 65.3 (25.0), respectively.

#### Distribution method

Half the standard deviation of the baseline SF-36-PFS scores is 10.7 (half of baseline SD of 21.4, *N* = 198) with 95% CI (9.7 to 11.9).

#### Anchor method

We used the CGI as the Anchor and analysed change in SF-36-PFS from baseline to 6-month follow-up for each response on the CGI. Table 2 displays the mean difference on the SF-36-PFS from baseline to 6-months follow-up for each level of the CGI. “A little better” on the CGI equates to a difference of 8.8 (95% CI 3.9 to 13.7, *n* = 67/198).

**Table 2:** Mean change in SF-36-PFS scores from baseline to 6-month follow-up for each level of the CGI response

Clinical Global Impression at 6 months’ follow-up	SF-36-PFS change between baseline and 6-months’ follow-up			
	Median (IQR)	Mean (95% CI)	SD	n
Very much better	30 (20, 40)	33.1 (25.1, 41.0)	19.3	25
Much better	20 (5, 35)	20.1 (14.8, 25.5)	22.3	69
A little better	10 (−5, 20)	8.8 (3.9, 13.7)	20.1	67
No change	0 (−12, 15)	−0.9 (−11.9, 10.1)	18.2	13
A little worse	7 (−10, 10)	3.8 (−9.9, 17.6)	22.7	13
Much worse	−15 (−20, 5)	−6.8 (− 20.5, 6.9)	17.8	9
Very much worse	−25 (−40, −10)	−25.0 (− 215.6, 165.6)	21.2	2

### Qualitative results

Twenty-one children with CFS/ME participated: 16 females (76.2%) with a mean age of 14.4 years. Twenty mothers and two fathers were also interviewed.

Eighteen out of 21 of the participants’ and their parents/carers went through the SF-36-PFS during the interview. At interview, one child had recovered and did not take part. Two copies of the SF-36-PFS were unavailable for analyses. On average, parents/ carers rated their child as less disabled (median = 50), in comparisons to the young person’s rating (median = 37.5). The distribution of change was highly skewed. The median minimal improvement in the SF-36-PFS was 10.

A number of participants had difficulty understanding the concept of MCID and the purpose of the exercise. Eight children required clarification or further explanation before they could complete the exercise due to confusion about terms used or what was being asked of them. Meanwhile, parents were in general more receptive, giving longer, more detailed responses.

### “Cause it’s small things”

During discussions, participants emphasised the importance of basic mobility as a marker of meaningful improvement. Participants typically felt walking (100 yards or half a mile) and climbing one flight of stairs was important as this level of mobility was seen as necessary for the basic and essential daily tasks like being able to move around their own home.

“*Yeah, I’ve got, erm, my bedroom upstairs and my brother’s bedroom is up-upstairs… So you’ve got to go up those stairs*” (Child).

Parents and children talked about how small changes were important

*“If like the climbing several flights of stairs went not limited at all, would be a good, quite small change”* (Child)

Participants perceived meaningful improvement as that which enabled them to carry out routine basic daily activities related to roles and relationships valuable to them, with emphasis often being placed on involvement in family and social activities:

*“So you can help around the house and, and do things with people.”* (Child).

Participants commonly expressed a desire to be able to carry out these activities without experiencing physical discomfort such as pain.

*“Being able to lift and carry stuff, and not ache afterwards.”* (Child).

### Accepting some level of limitation (in vigorous activity and walking long distances)

Participants talked about acceptance of some level of limitation. This was particularly true for the domain of vigorous activity. Although some participants would like to participate in exercise, they did not see this as an essential marker of improvement.

*“That’s it really, I mean I’m happy to just sort of get on with it if it’s a little bit limited, I can just deal with it*” (Child).

“*‘Cause, I mean, you don’t have to run, that’s not really a big thing.*” (Child).

Participants did not need complete recovery in walking more than a mile and vigorous activities, as this was seen as more of a luxury than a necessity of daily functioning. Younger children talked more about wanting to return to more vigorous activities such as sports. This may be because P.E and afterschool clubs are important opportunities for social interaction for young children. One participant did state that vigorous activity was important. They felt that their condition had caused weight gain and that being able to vigorous activity could be a way to manage this.

### Limitation of the SF-36-PFS and the MCID interview

The interviews revealed the limited scope of the SF-36-PFS questions. The SF-36-PFS asks about specific activities but not all aspects of SF-36-PFS were relevant to participants and in some cases, the relevance changed from day to day:

*“But yeah I think some of the things on here I’ve never really had a problem with doing anyway, like bathing, undressing myself never really has been a problem. Bending or stopping like there’s never really been a problem with that, like I’ve aches and pains but it’s never stopped me from doing any of those, so yeah.”* (Child).

*“…it’s so variable day to day that you can’t pigeonhole it in those little things like, do you know what I mean? I don’t know if that’s the answer you want, but that is the answer I feel is right…”* (Parent).

### Triangulating the findings

To triangulate the findings, we calculated the mean of the scores [31] from the distribution method (10.90), the anchor method (8.99) and the qualitative interviews (10) to determine an MCID of 10.

## Discussion

This is the first study to calculate the minimally clinically important difference (MCID) of the SF-36-PFS for young people with CFS/ME. It is also the first study to calculate the MCID of the SF-36-PFS for children with a chronic disease. We used three different methods, which suggested that a MCID of 10 was appropriate (distribution method = 10.7, anchor method = 8.8 and qualitative interviews = 10). The qualitative data enriches this finding, indicating that small changes in physical function can lead to important improvements in valued social and family function. Patients and parents are positive about improvement even in the presence of persisting symptoms.

### Strengths and limitations

The main strength is that we used three different methods to define the MCID of the SF-36-PFS in this patient group, which increases our confidence in the result. Using multiple quantitative methods increases confidence in the accuracy of the findings, and qualitative methods ensures the MCID is clinically relevant. The sample sizes for the statistical methods were reasonably large. The overall proportion of children who provided 6-month follow-up data was relatively low, but we would not expect this to bias the relationship between follow-up SF-36-PFS scores and CGI levels or to yield a wider or narrower standard deviation for baseline SF-36-PFS scores.

Qualitative interviews included both young people and their parents, because children and parents do not necessarily share similar views about the impact of illness [32]. All participants received their diagnosis from a large specialist paediatric CFS/ME service, and our sample was representative of patients attending the service. Since a number of interviews were conducted with the child’s parent(s) present, it is possible that parents may have influenced the child’s answer. Participants were recruited from one specialist service, the results may not be generalisable to other services. We did not interview children who were under the age of 12, severely affected (house or bed bound) and therefore we cannot extrapolate results to these patient groups.

We only looked at the MCID of one scale, the SF-36-PFS, which captures physical disability. This study has not considered MCID for scales that capture other aspects of the illness, such as symptoms of fatigue and pain. However, the SF-36-PFS in an important measure as young people with CFS/ME feel that a lack of social participation and low mood is secondary to the symptoms and physical disability they experience [33, 34]. Further, it has been used in studies for paediatric CFS/ME in the UK, Australia and the Netherlands [14, 17, 35–37].

### Context of previous literature

We were unable to compare our results with other paediatric studies, because we could not find published data on the MCID for the SF-36-PFS. However, our results are consistent with a Delphi consensus, which suggested an MCID of 10 for asthma and heart disease [18]. The range in adult studies investigating the MCID in different conditions with different methodology is wide (3.25–20.40) [18–20].

Our qualitative findings suggested that small changes are important because they enabled greater function, even with the persistence of symptoms. This is consistent with the views of patients with COPD who acknowledged that a large improvement on the Breathlessness Diary (BD) measure may not be a realistic goal of treatment, and reported that a 1-point step-change would be considered “dramatic” [8].

## Conclusions

An MCID of 10 should be used in paediatric CFS/ME treatment trials and observational studies for the SF-36-PFS. Clinicians should remember that relatively small changes in physical function are worthwhile for patients and their families as this can lead to a reduction in social and family limitations.

Further research is needed to define the MCID on measures which capture other aspects of CFS/ME, such as fatigue and pain measures.

## Additional file

Additional file 1: Extract from Topic Guide: Outcomes in Paediatric CFS/ME (PDF 65 kb)

## Abbreviations

CFS/ME: Chronic Fatigue Syndrome / Myalgic Encephalomyelitis
CGI: Clinical Global Impression Scale
MCID: Minimal Clinically Important Difference
SF-36-PFS: The SF-36 physical function sub-scale
