# Correction: Defining the minimally clinically important difference of the SF-36 physical function subscale for paediatric CFS/ME: triangulation using three different methods

**DOI:** 10.1186/s12955-022-02078-7

**Published:** 2023-02-18

**Authors:** Amberly Brigden, Roxanne M Parslow, Daisy Gaunt, Simon M Collin, Andy Jones, Esther Crawley

**Affiliations:** 1 Population Health Sciences, Centre for Academic Child Health, Bristol Medical School, University of Bristol, 1-5 Whiteladies Road, Bristol, BS8 1NU UK; 2 Bristol Randomised Trials Collaboration, Population Health Sciences, Bristol Medical School, University of Bristol, Bristol, BS8 2PS UK

**Correction: Health and Quality of Life Outcomes (2018) 16:202** 10.1186/s12955-018-1028-2

The original article [1] required an amendment to the Quantitative Data statement in the Ethics Declarations section. The statement has since been updated.
