# Supplementary material for: Defining the minimally clinically important difference of the SF-36 physical function subscale for paediatric CFS/ME: triangulation using three different methods
Source: Health Qual Life Outcomes. 2018 Oct 19;16:202. doi: 10.1186/s12955-018-1028-2 (PMC6194701; doi:10.1186/s12955-018-1028-2)
Supplement: Supplementary file 1 — Extract from Topic Guide: Outcomes in Paediatric CFS/ME (PDF 65 kb) [file 12955_2018_1028_MOESM1_ESM.pdf]

Extract from Topic Guide: Outcomes in Paediatric CFS/ME

Minimal Clinically Important Difference (MCID) Topic Guide

Young Person Topic Guide

Minimal Clinically Important Difference (MCID)

MCID= change in the measure that a child thinks makes a difference to their lives.

Get young person to fill out SF36-PFS for how they feel now. Take a red pen and get the young person to mark the SMALLEST AMOUNT the responses would have to change to make a difference to them.

☐ **What would be the SMALLEST AMOUNT these would have to change by for you to feel like treatment has made a difference to you?**

Prompts: How many responses would have to improve, by how much…

Parent Topic Guide

Minimal Clinically Important Difference (MCID)

MCID= change in the measure that a child thinks makes a difference to their lives.

Present parent with young person's completed SF36-PFS response. Take a red pen and get parent to mark the SMALLEST AMOUNT the responses would have to change to make a difference to them.

We just asked [child] to complete the questionnaire and these are the answers they gave.

☐ **What would be the SMALLEST AMOUNT these would have to change by for you to feel like treatment has made a difference?**

Prompts: How many responses would have to improve, by how much…
